# Effectiveness of different disinfection techniques of the root canal in the elimination of a multi-species biofilm

**DOI:** 10.4317/jced.56000

**Published:** 2019-11-01

**Authors:** Abel Teves, Daniel Blanco, Mario Casaretto, John Torres, Debora Alvarado, David E. Jaramillo

**Affiliations:** 1 Universidad Inca Garcilaso de la Vega; 2 Universidad Peruana Cayetano Heredia; 3 Universidad Nacional Mayor de San Marcos; 4 Universidad Catolica Santo Toribio de Mogroviejo; 5 Professor of Endodontics University of Texas Health Science Center at Houston

## Abstract

**Background:**

The purpose of the study was to evaluate the effectiveness of different root canal disinfection techniques in the elimination of a multi-species biofilm from inside the root canal.

**Material and Methods:**

Fifty mandibular first premolars were used in the present study, standardized to 11mm of root length, and instrumented with a reciprocation system Reciproc, (VDW GmbH, Munich, Germany) to a #50. Longitudinally sectioned halves of the roots were obtained and washed with NaOCl 4%, EDTA 17% and 5% sodium thiosulfate, and sterilized by autoclaving for 15 minutes at 121°C. A multi-species biofilm broth was developed with three strains of bacteria under laboratory conditions: *Enterococcus faecalis* ATTC 29212, *Eikenella corrodens* ATTC 23834, *Streptococcus anginosus* ATTC 33397. Roots were autoclaved and transferred to the broth for 4 days and then were subjected to either disinfection with sodium hypochlorite 4% and XP-endo Finisher (FKG Dentaire, La Chaux-de-Fonds, Switzerland) or chlorhexidine 2% with and without activation with XP-endo Finisher (FKG Dentaire, La Chaux-de-Fonds, Switzerland).

**Results:**

The evaluations of the biofilm elimination showed results that indicate that the 4% sodium hypochlorite group with positive pressure irrigation presented significant differences with the group that had irrigation with sodium hypochlorite activated with XP-endo Finisher and the chlorhexidine groups to 2% (*P*<0.05).

**Conclusions:**

Chlorhexidine 2% activated with the XP-endo Finisher does not exert elimination or improved cleaning effect on the multi-species biofilm. Activation of sodium hypochlorite 4% improved the elimination of the multi-species biofilm.

** Key words:**Biofilm, multispecies, chlorhexidine, sodium hypochlorite.

## Introduction

The main cause of pulpal and periapical infection is the presence of bacteria inside the pulp and root cavity (1). One of the root canal therapy objectives is the elimination of the microflora and inflamed pulp tissue of the root canal (2) as well as avoiding and preventing apical periodontitis (3). The bacterial biofilm is made up of individual cells and micro-colonies, all embedded in a highly hydrated exopolymer matrix (4). Chávez de Paz (5) isolated 248 bacterial strains in root canals where the most prevalent was Gram-positive bacteria in 85%, and he determined that the Gram positive bacteria in the root canal system can survive even after the cleaning and disinfection of the root canal. The microorganisms live protected from environmental threats by the structure of the biofilm (6) so persistent infections depend, not in the robustness of the organisms in the infected sites, but in their ability to adapt their physiology to the new environmental conditions established by the treatment (7). Sterilization of the root canal system is practically impossible to achieve even with current instruments, irrigation solutions and different irrigation techniques. Therefore, what is sought in the therapy of root canals is the reduction of the bacterial load to levels compatible for the healing of periradicular tissue (8). The use of chemical agents during instrumentation for a complete disinfection of the entire root canal system is critical to the success of endodontic treatment. Irrigation is complementary to the instrumentation to facilitate and contribute to the elimination of microorganisms and pulp tissue (9). Sodium hypochlorite (NaOCl) is a proteolytic agent with a large non-specific bactericidal effect and is the main irrigation solution in endodontic therapy (10). Chlorhexidine is a solution with strong bactericidal effect (11), but its ability to dissolve organic matter and biofilm is limited as mentioned by del Carpio (12). The activation of the irrigant has been shown to improve the effect of irrigation (13). Ultrasonic irrigation of the root canal system has been studied extensively with good results. However, there is disagreement about the relative efficacy of the ultrasonic activation to completely eradicate the microorganisms (14).

The XP Endo Finisher (FKG Dentaire, La Chaux-de-Fonds, Switzerland) is an instrument that has been recently introduced. It was designed to have increased contact in greater surface of the root canal wall and an optimal cleaning of the root canal system (15). Therefore, in the present study the objective of evaluating the effectiveness of different root canal disinfection techniques in the elimination of multi-species biofilm is proposed.

## Material and Methods

All patient had given consent form and approval was granted from the institutional ethics committee at Universidad Inca Garcilaso de la Vega.

-Preparation of specimens

Fifty recently extracted mandibular first premolars were used. The clinical crowns were removed and the roots were standardized to a length of 11mm. After mounting the teeth in a Zetaplus silicone system (Zhermack, Badia Polesine RO, Italy) to produce silicone-based positioning system. Roots were instrumented with Reciproc R50 (VDW GmbH, Munich, Germany) and sectioned longitudinally. Notches were made with a ½ round carbide low speed bur at 3, 5 and 8 mm half way in between the root canal and the cementum, to be able to consistently locate the evaluation areas of the root canal. The root halves were washed with NaOCl 4% and EDTA 17% (Maquira, Parana, PR, Brazil). After using 5% sodium thiosulfate to inactivate the NaOCl, the teeth were sterilized and stored until use.

-Biofilm growth

A multi-species biofilm model was developed with three bacterial strains under laboratory conditions: *Enterococcus faecalis* ATTC 29212, *Eikenella Corrodens* ATTC 23834, *Streptococcus anginosus* ATTC 33397. Lyophilized strains were rehydrated in Brain Heart Infusion Broth (Sigma aldrich, Sao Paulo, Brazil) enriched with Hemin (Sigma aldrich, Sao Paulo, Brazil) (15 mg / L), in a anaerobic jar GasPack (BD, New York, USA) with Anaerocult A (Merck Millipore, Massachusetts, USA). An inoculum of 100 μl of each of the strains was used when they were in an optical density of 0.5 Macfarland, these were inoculated in 3 ml of BHI broth with Hemina and the teeth were incubated for 72 hours.

-Experimentation procedure

After the infection process, the samples were removed from the media and the external root surface was cleaned with 4% NaOCl and inactivated with 5% sodium thiosulfate. The root halves were assembled in the silicone-based positioning system and then divided into the following evaluation groups containing 10 samples in each:

Group 1: NaOCl 4% + irrigation with positive pressure (n = 10)

Group 2: NaOCl 4% + agitation with XP-endo Finisher (n = 10)

Group 3: Chlorhexidine 2% + irrigation with positive pressure (n = 10)

Group 4: Chlorhexidine 2% + agitation with XP-endo Finisher (n = 10)

Group 5: distilled water + irrigation with positive pressure (n = 10)

A primary irrigation phase was done in all samples using a 30ga NaviTip needle (Ultradent Products, Inc., South Jordan, UT, USA) attached to a 10 ml syringe of NaOCl 4% situated at 2 mm from the working length. 1ml was injected with a flow rate of 1ml/10s. The irrigant was left in the canal for a period of 2 minutes and this step was repeated for a second time for a total of 2 ml. The final irrigation protocols were then accomplished:

Group 1: After the primary irrigation with the aforementioned method, the final irrigation was performed using a NaviTip needle 30 ga in a 10ml syringe with 4 % sodium hypochlorite. The needle was inserted at 2 mm of the working length, 3 cycles of irrigation were carried out passively with a flow of 1 ml / 10s and then the irrigant was left for a period of 20 seconds between each cycle. In total, 3 ml was used per procedure.

Group 2: The X-Smart Plus motor (Densply Sirona Endodontics, Ballaigues, Switzerland) was used with the programming of 800 rpm and 1 N of torque with the final cleaning file XP-Endo Finisher (FKG Dentaire, La Chaux de Fonds, Switzerland). This file was placed in the root canal heating irrigation solution to 37 ° C so to allow the instrument to reach the austenitic phase. Previoulsy, the working length was fixed with the plastic tube, after cooling with EndoFrost (Coltene, New York, NY, USA). The plastic tube was removed and introduced into the root canal at the working length of the tooth, the kinematics consisted of movements from 7 to 8 mm input and output, and this procedure lasted 60 seconds.

Group 3: In this group 2% Chlorhexidine (Maquira, Paraná, PR, Brazil) was used by with positive pressure irrigation, which consisted of irrigating with a 10 ml syringe. and NaviTip needle 30 ga at 2 mm short from working length. Three passive irrigation cycles were performed with a flow of 1 ml / 10s and then the irrigant was left in the canal for a period of 20 seconds between each cycle. The total irrigant per group was 3 ml.

Group 4: Irrigation was carried out with 2% chlorhexidine in a 10 ml syringe and NaviTip 30 ga needle. The irrigant was agitated with the XP-endo Finisher file using the protocol described in group 3.

Group 5: This was the control group and was irrigated with sterile distilled water with 10 ml syringe and 30 ga NaviTip needle at 2 mm working length with an irrigant flow of 1ml / 10 s, 3 cycles of this were repeated for a total of irrigant 3 ml.

-Processing of samples in scanning electron microscopy

After performing the disinfection process, roots were fixed in 4% formalin, followed by 2.5% glutaraldehyde (Merck USA, California, USA) and dehydration with succession of alcohols. Then samples were individually mounted in aluminum stubs Once done, these were taken to the desiccator at a critical point and later on to a sputtering with gold. The samples were mounted on the scanning electron microscope (FEI, Inspect S50, Hillsboro, OR, USA), with the parameters of the equipment set at 10 KV and 2400 X of magnification for observation. The images were then digitized.

-Image processing

The scanning electron microscopy images were taken and passed to an image processing program Photoshop SC (Adobe Systems Incorporated, California, USA) in which the areas of possible biofilm formations were selected, then the images were processed with ImageJ 1.50 software (National Institutes of Health, Maryland, USA) with which the results were obtained.

-Statistical analysis

**Figure 1:**
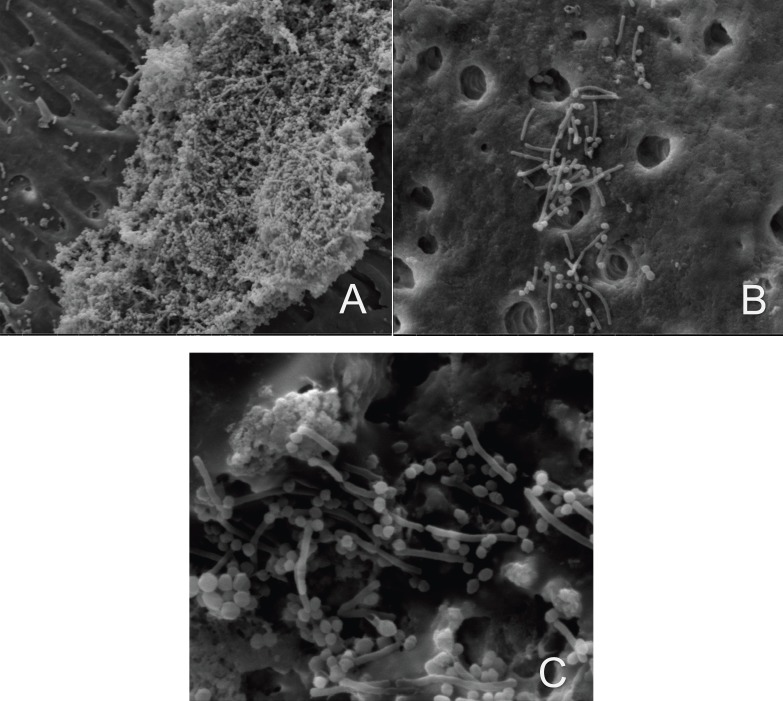
Scanning electron microscopy images showing the formation of a multi-species biofilm with three bacteria *Enterococcus faecalis* ATTC 29212, *Eikenella Corrodens* ATTC 23834, *Streptococcus anginosus* ATTC 33397 A) 4000x, B) 10000x, C) 20000 x increase.

**Figure 2:**
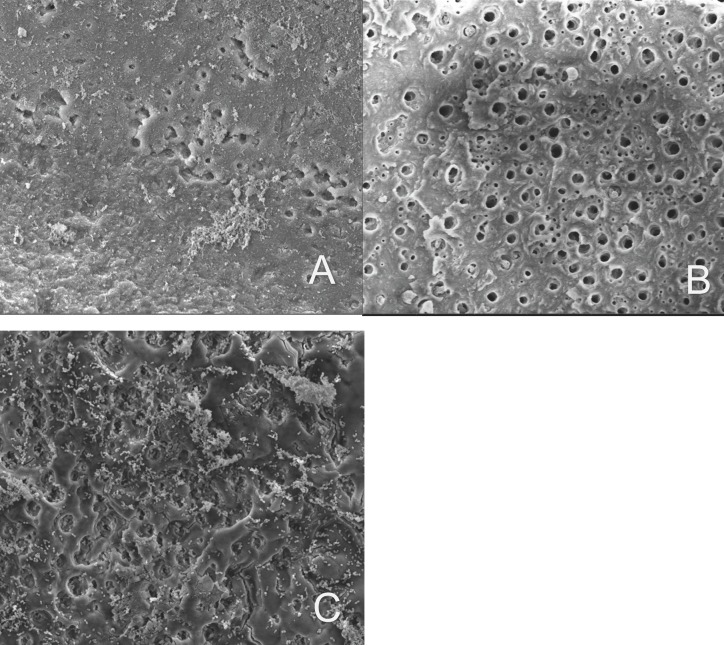
Scanning electron microscopy images showing the groups that were evaluated, all images were observed with an increase of 2400x, a) Irrigation with NaoCl 4% with positive pressure, b) NaOCl 4% with XP-endo finisher, c) Chlorhexidine 2% with irrigation with positive pressure.

**Figure 3:**
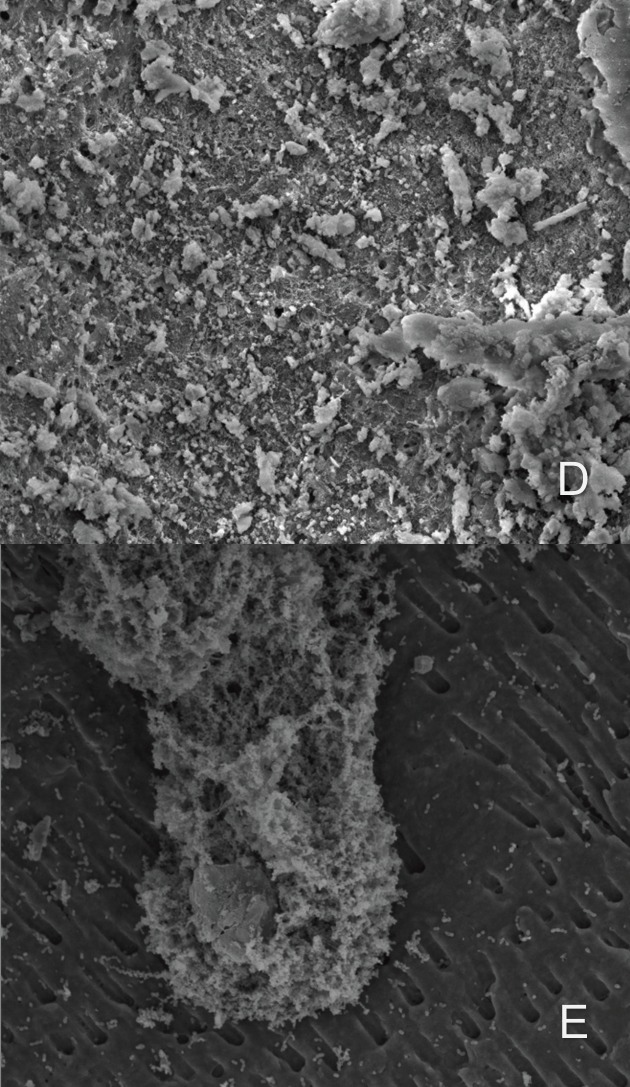
Scanning electron microscopy images showing the groups that were evaluated, all images were observed with an increase of 2400x, D) Chlorhexidine 2% with XP-endo Finisher, E) distilled water with irrigation with positive pressure.

After obtaining the data through image processing, the one-way variance analysis (ANOVA) test was carried out, (Figs. 1-3).

## Results

The evaluations of the biofilm elimination showed results that indicate that the 4% sodium hypochlorite group with positive pressure irrigation presented significant differences with the group that had irrigation with sodium hypochlorite activated with XP-endo Finisher and the chlorhexidine groups to 2% (*P* <0.05).

Significant differences were found in the irrigation group with sodium hypochlorite 4% with activation, compared to the groups with and without activated chlorhexidine (*P* <0.05).

2% chlorhexidine irrigated with a syringe showed no significant difference with the same irrigant group activated with XP-endo Finisher (*P*> 0.05).

There is no significant difference in the cleaning of the multi-species biofilm between the control group with distilled water and the groups with 2% chlorhexidine (*p*> 0.05), ( Table 1).

**Table 1:**
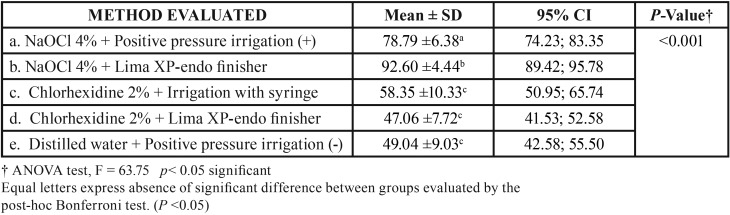
Distribution of means of biofilm elimination among all the techniques evaluated for root canal disinfection.

## Discussion

The present study analyzed different current irrigation techniques for the evaluation of the elimination of the multi-species biofilm. These included protocols with sodium hypochlorite at 4% and chlorhexidine at 2%, in both cases with positive pressure irrigation and activated with Xp-endo Finisher. Specimens were evaluated using scanning electron microscopy (16,17). The evaluation of the presence of biofilm with scanning electron microscopy is a technique that allows evaluating in a clear way the absence or presence of a biofilm on a surface.

It has been shown that the resistant polymicrobial infections where Gram-positive organisms predominate, such as streptococci and enterococci, are the cause of endodontic failure. Chávez de Paz (5) found that Gram-positive organisms persisted after several sessions of irrigation and medication, and determined that they are very difficult to eliminate when lodged in the root canal. In the present study three types of bacteria were used, two of which were Gram positive (*Enterococcus faecalis* ATTC 29212 and *Streptococcus anginosus* ATTC 33397). The Gram-negative organisms are not excluded from also being present in the root canal in a considerable proportion. Rocas (18), in a bacterial isolation investigation, found *Eikenella Corrodens* in 63% as the second microorganism in greatest quantity present in chronic apical periodontitis. This microorganism was also used as a bacterium next to the two already mentioned in the formation of a multi-species biofilm and was developed under laboratory conditions in partial anaerobiosis cultured at 37 ° C for 3 days. Endodontic disinfection presents two major challenges: the anatomical and the microbiological (19). For this, it uses different irrigating substances that seek to eliminate pulp tissue, smear layer and a biofilm remaining from areas not touched by the instrument (20). Sodium hypochlorite in its different concentrations (21) as well as chlorhexidine are highly studied irrigants with respect to their susceptibility (22) in the elimination of the biofilm (12). In molecular studies with PCR (8), we find both positive and negative results depending on the methodology and the research objective, Most of these studies are carried out under irrigation conditions with positive pressure.

Our results determine that sodium hypochlorite 4% without being activated with the XP-endo finisher produces a greater cleaning of the dentin surface than chlorhexidine 2% in the same condition without any activation of the irrigant. The activation of the irrigant using ultrasonics is a widely used protocol that improves the irrigation of the root canals by the acoustic effects (13). In their study, Ordinola *et al.* (23) using an intraoral biofilm, showed ultrasonics favored biofilm removal from the surface of infected dentin, compared to Endo-activator (sonic) agitation and positive pressure. Currently, new alternatives are being sought for cleaning the root canal system. Recently, the XP-endo finisher was introduced to the market, this is a new instrument for final finishing and cleaning of dentinal walls. It presents promising studies in the cleaning of the smear layer similar to ultrasonic irrigation (24). The research that aims to evaluate the dentinal cleanliness contaminated with a salivary biofilm that compares ultrasonically activated irrigation and the XP-Endo finisher file, as in the Pingping Bao study, demonstrated better results with XP-endo finisher, which is why we decided to use this new instruments for our research.(25) Azim (15) found that irrigation activated with the XP - Endo Finisher had better results in the elimination of bacteria in dentinal tubules up to 50 μm deep than conventional irrigation, results that resemble ours but with different methodology. Chlorhexidine has no organic tissue dissolving effect (26), although activating the irrigation solution may have some effect on the biofilm removal or disruption. Shen (14) found that when 2% chlorhexidine activated ultrasonically, it did not produce biofilm removal.

Chlorhexidine activated with XP-endo finisher could be an alternative to cover the cleansing deficit of organic tissue. The present study determined that chlorhexidine 2% activated with the XP-Endo finisher file did not eliminate biofilm from the dentin walls (results equal to the control and irrigation group with positive pressure). The mechanical cleaning effect of the XP-endo finisher itself does not seem to have effects of elimination of the biofilm, its effect is enhanced when combined with an irrigant that has the capacity to dissolve organic tissue. The present study aims to give parameters on what final irrigation protocol allows us to obtain a biofilm-free dentine surface and to be able to extrapolate it to our daily clinical practice so that our procedures in patients are based on scientific evidence and thus are more predictable and ensure a higher success rate.

## Conclusions

The irrigation technique of 4% sodium hypochlorite with XP-Endo Finisher activation eliminated more biofilm than the irrigation technique of sodium hypochlorite at 4% positive pressure.

The technique of irrigation with 4% sodium hypochlorite with syringe eliminated more biofilm than the techniques with 2% chlorhexidine.

The irrigation technique with 2% Chlorhexidine with positive pressure eliminated the same amount of biofilm as the irrigation technique of 2% chlorhexidine with XP-endo Finisher.

The control group had the same results in biofilm elimination as the 2% chlorhexidine groups with syringe irrigation and 2% chlorhexidine with XP-endo Finisher

The irrigation technique with 4% sodium hypochlorite with positive pressure had better results than the irrigation of distilled water with positive pressure in a multispecies biofilm.

## References

[1] Kakehashi S, Stanley HR, Fitzgerald RJ (1965). The effects of surgical exposures of dental pulps in germ-free and conventional laboratory rats. Oral Surgery, Oral Medicine, Oral Pathology and Oral Radiology.

[2] Trope M, Bergenholtz G (2002). Microbiological basis for endodontic treatment: Can a maximal outcome be achieved in one visit?. Endodontics: a microbial issue. Endodontic Topics.

[3] Nair PNR (2006). On the Causes of Persistent Apical Periodontitis-a Review. Internacional Endodontic Journal.

[4] Costerton JW, Cheng KJ, Geesey GG, Ladd TI, Nickel JC, Dasgupta M (1987). Bacterial biofilms in nature and disease. Annual Review of Microbiology.

[5] Chávez De Paz LE, Dahlén G, Molander A, Möller Å, Bergenholtz G (2003). Bacteria recovered from teeth with apical periodontitis after antimicrobial endodontic treatment. Internacional Endodontic Journal.

[6] Lewis K (2001). Riddle of biofilm resistance. Antimicrobial Agents and Chemotherapy.

[7] Chávez de Paz LE (2007). Redefining the Persistent Infection in Root Canals: Possible Role of Biofilm Communities. Journal of Endodontics.

[8] Rôças IN, Provenzano JC, Neves MAS, Siqueira JF (2016). Disinfecting Effects of Rotary Instrumentation with Either 2.5% Sodium Hypochlorite or 2% Chlorhexidine as the Main Irrigant: A Randomized Clinical Study. Journal of Endodontics.

[9] Shen Y, Stojicic S, Haapasalo M (2011). Antimicrobial efficacy of chlorhexidine against bacteria in biofilms at different stages of development. Journal of Endodontics.

[10] Mohammadi Z (2008). Sodium hypochlorite in endodontics: an update review. International Dental Journal.

[11] Mohammadi Z, Jafarzadeh H, Shalavi S (2014). Antimicrobial efficacy of chlorhexidine as a root canal irrigant: a literature review. Journal of Oral Science.

[12] Del Carpio-Perochena AE, Bramante CM, Duarte MAH, Cavenago BC, Villas-Boas MH, Graeff MS (2011). Biofilm dissolution and cleaning ability of different irrigant solutions on intraorally infected dentin. Journal of Endodontics.

[13] Lucas van der Sluis WM, Vogels MPJM, Verhaagen B, Macedo R, Wesselink PR (2010). Study on the Influence of Refreshment/Activation Cycles and Irrigants on Mechanical Cleaning Efficiency During Ultrasonic Activation of the Irrigant. Journal of Endodontics.

[14] Shen Y, Stojicic S, Qian W, Olsen I, Haapasalo M (2010). The Synergistic Antimicrobial Effect by Mechanical Agitation and Two Chlorhexidine Preparations on Biofilm Bacteria. Journal of Endodontics.

[15] Azim AA, Aksel H, Zhuang T, Mashtare T, Babu JP, Huang GTJ (2016). Efficacy of 4 Irrigation Protocols in Killing Bacteria Colonized in Dentinal Tubules Examined by a Novel Confocal Laser Scanning Microscope Analysis. Journal of Endodontics.

[16] Chávez de Paz LE, Bergenholtz G, Svensäter G (2010). The Effects of Antimicrobials on Endodontic Biofilm Bacteria. Journal of Endodontics.

[17] George S, Kishen A, Song KP (2005). The role of environmental changes on monospecies biofilm formation on root canal wall by Enterococcus faecalis. Journal of Endodontics.

[18] Rôças IN, Siqueira JF (2008). Root canal microbiota of teeth with chronic apical periodontitis. Journal of Clinical Microbiology.

[19] Kishen A (2010). Advanced therapeutic options for endodontic biofilms. Endodontic Topics.

[20] Peters OA (2004). Current challenges and concepts in the preparation of root canal systems: A review. Journal of Endodontics.

[21] Grossman LI, Meiman BW (1982). Solution of pulp tissue by chemical agents. Journal of Endodontics.

[22] Sassone LM, Fidel RAS, Murad CF, Fidel SR, Hirata R (2008). Antimicrobial activity of sodium hypochlorite and chlorhexidine by two different tests. Australian Endodontic Journal.

[23] Ordinola-Zapata R, Bramante CM, Aprecio RM, Handysides R, Jaramillo DE (2014). Biofilm removal by 6% sodium hypochlorite activated by different irrigation techniques. International Endodontic Journal.

[24] Leoni GB, Versiani MA, Silva-Sousa YT, Bruniera JFB, Pécora JD, Sousa-Neto MD (2017). Ex vivo evaluation of four final irrigation protocols on the removal of hard-tissue debris from the mesial root canal system of mandibular first molars. International Endodontic Journal.

[25] Bao P, Shen Y, Lin J, Haapasalo M (2017). In Vitro Efficacy of XP-endo Finisher with 2 Different Protocols on Biofilm Removal from Apical Root Canals. Journal of Endodontics.

[26] Basrani B, Lemonie C (2005). Chlorhexidine gluconate. Australian Endodontic Journal.

